# Trait mindfulness during pregnancy and perception of childbirth

**DOI:** 10.1007/s00737-020-01062-8

**Published:** 2020-09-08

**Authors:** Lianne P. Hulsbosch, Myrthe G. B. M. Boekhorst, Eva S. Potharst, Victor J. M. Pop, Ivan Nyklíček

**Affiliations:** 1 Center of Research in Psychological and Somatic disorders (CoRPS), Department of Medical and Clinical Psychology, Tilburg University, Box 90153, 5000 LE Tilburg, The Netherlands; 2 UvA Minds, Academic Outpatient (Child and Adolescent) Treatment Center, University of Amsterdam, Amsterdam, The Netherlands; 3 Research Institute of Child Development and Education, University of Amsterdam, Amsterdam, The Netherlands

**Keywords:** Childbirth experience, Delivery, Perception, Mindfulness, Pregnancy

## Abstract

Women’s subjective childbirth experience is a risk factor for postpartum depression and childbirth-related posttraumatic stress symptoms. Subjective childbirth experience is influenced not only by characteristics of the childbirth itself but also by maternal characteristics. A maternal characteristic that may be associated with a more positive childbirth experience is trait mindfulness. The current study aimed to assess this association and to assess whether trait mindfulness during pregnancy had a moderating role in the possible association between non-spontaneous delivery and perception of childbirth. A subsample of 486 women, participating in a longitudinal prospective cohort study (Holistic Approach to Pregnancy and the first Postpartum Year study), completed the Three Facet Mindfulness Questionnaire-Short Form at 22 weeks of pregnancy. Women completed the Childbirth Perception Scale and the Edinburgh Postnatal Depression Scale between 7 and 21 days postpartum. The mindfulness facets acting with awareness and non-reacting were significantly associated with a more positive perception of childbirth, after adjusting for covariates. Moderation analyses showed a significant interaction between acting with awareness and non-spontaneous delivery and non-judging and non-spontaneous delivery. Non-spontaneous delivery was associated with a more negative perception of childbirth for low/medium scores of acting with awareness and non-judging, but not for high scores on these mindfulness facets. Trait mindfulness during pregnancy may enhance a positive perception of childbirth. Because this is among the first studies examining the association between maternal dispositional mindfulness and perception of childbirth, future research is needed to confirm the results of the current study.

## Introduction

Even though childbirth is usually experienced as painful and overwhelming, most women look back at it positively. However, some women perceive childbirth as a very stressful or even psychologically traumatic event (Boorman et al. 2014). Women’s subjective experience of childbirth is a risk factor for maternal mental health challenges, such as postpartum depression (Bell and Andersson 2016) and childbirth-related posttraumatic stress symptoms (Ayers et al. 2016; Dekel et al. 2017; Grekin and O’Hara 2014). In turn, this can have consequences for child development, such as cognitive and emotional development (Beck 1998; Cook et al. 2018; Garthus-Niegel et al. 2013; Goodman et al. 2011; O’Hara and McCabe 2013). Furthermore, postpartum depression negatively affects parenting and the mother-child interaction (O’Hara and McCabe 2013).

Non-spontaneous delivery, including induced labor, instrumental vaginal delivery, and unplanned Caesarean section, has been associated with a more negative childbirth experience (Lobel and DeLuca 2007; Waldenstrom 1999; Waldenstrom et al. 2004). In a study by Shetty et al. (2005), it was found that even though the majority of women who had induced labor were happy with the decision, lower labor satisfaction rates were found in women with induced labor compared with that in women with spontaneous deliveries. Lower labor satisfaction was shown to be associated with the duration of the induction, mode of administration of the inducing agent, and more vaginal examinations (Shetty et al. 2005). It should be noted, however, that rates of induction vary across western countries with lower rates in northern countries of Europe (Scandinavian, The Netherlands) and higher rates in southern countries. Moreover, primiparity, inaccurate expectations, lack of midwife and partner support, and anxiety and fear for labor during pregnancy as well as pain and lack of control during labor have been related to a more negative perception of childbirth (Hodnett 2002; Waldenstrom 1999; Waldenstrom et al. 2004).

Mindfulness may facilitate effective coping with aversive experiences during childbirth, such as feelings of not being in control, pain, anxiety, and unexpected (medical) events, by means of having full attention to the present moment experience, and relating to the present moment differently, namely with an open, non-judgmental, and accepting attitude (Bishop et al. 2004). Mindfulness has been conceptualized as both a state and a trait (Baer et al. 2006; Brown and Ryan 2003). A state of mindfulness can be seen as a psychological process, is variable over time, and is dependent on the specific situation (Bishop et al. 2004; Tanay and Bernstein 2013). State mindfulness can be practiced during mindfulness meditation. Trait mindfulness is one’s predisposition to be mindful in daily life. Increasing state mindfulness during meditation practice can increase trait mindfulness over time (Kiken et al. 2015), but without an intervention, trait mindfulness appears to be stable over time (Brown and Ryan 2003). Indeed, a test-retest reliability in a Dutch, mostly female, sample showed good stability of mindfulness across time (Veehof et al. 2011). Therefore, it is likely that without an intervention, trait mindfulness scores are relatively stable.

A possible factor that may be associated with a more positive childbirth experience is trait mindfulness. To our knowledge, no studies have assessed the association between trait mindfulness and a woman’s perception of her childbirth experience. Nonetheless, studies on mindfulness-based childbirth interventions have provided insight into the role of (state) mindfulness during childbirth. Recent studies have examined the effects of antenatal mindfulness-based childbirth education, finding positive effects on perceived stress, depressive symptoms, state of mind, childbirth self-efficacy, anxiety, and level of mindfulness, measured during pregnancy (Byrne et al. 2014; Duncan et al. 2017; Lonnberg et al. 2019; Pan et al. 2019; Warriner et al. 2018). Furthermore, change in mindfulness mediated the effects of the intervention on perceived stress, depressive symptoms, and state of mind (Lonnberg et al. 2019).

Maternal mindfulness may enhance a positive childbirth experience. Higher levels of mindfulness may reduce negative reactions due to unavoidable distressing events and improve effective coping with these events (Bergomi et al. 2013a). A qualitative study showed that women who took part in an antenatal mindfulness-based childbirth education program felt more in control during childbirth even when the childbirth did not happen as planned (Fisher et al. 2012). However, to our knowledge, no quantitative studies examined the effect of maternal dispositional mindfulness on subjective childbirth experience.

Besides a possible direct association between maternal mindfulness and subjective childbirth experience, mindfulness may also protect against the adverse effects of a non-spontaneous delivery. A study by Nyklíček et al. (2015) found a buffering role of mindfulness against the negative associations between disability and psychological distress. Therefore, the current study aimed to assess two ways in which trait mindfulness during pregnancy may be associated with a positive perception of childbirth. Firstly, the current study aimed to assess whether trait mindfulness during pregnancy was associated with a positive perception of childbirth after adjustment for relevant obstetric and psychological covariates. Secondly, the current study aimed to assess whether trait mindfulness during pregnancy had a moderating role in the possible association between non-spontaneous delivery and perception of childbirth. It was hypothesized that maternal dispositional mindfulness could serve as a buffer against a more negative perception of childbirth, especially in the case of non-spontaneous delivery.

## Materials and methods

### Procedure

The current study is part of the Holistic Approach to Pregnancy and the first Postpartum Year (HAPPY) study, a large prospective cohort study among pregnant women (Truijens et al. 2014a). Dutch speaking white women (*N* = 2269) were recruited by 17 community midwife practices in the south of The Netherlands during their first antenatal visit, from January 2013 to September 2014. Exclusion criteria were multiple pregnancies, severe psychiatric disorder (e.g., schizophrenia, borderline personality disorder, and bipolar disorder), and/or a documented history of chronic disease (e.g., diabetes and thyroid dysfunction). The HAPPY study was approved by the ethical committee of Tilburg University (protocol number EV-2012.25) and reviewed by the Medical Ethics Committee of the Máxima Medical Centre Veldhoven. All women provided written informed consent.

### Participants

Only a subsample of women from the HAPPY study cohort was asked to complete a questionnaire measuring trait mindfulness at 22 weeks of pregnancy (*N* = 991); these were women who were included in the cohort between March 2013 and December 2013. A subsample of 1225 women completed a questionnaire about how they experienced the childbirth between 7 and 21 days postpartum. This resulted in a subsample of 523 women who completed both questionnaires. Women with a primary Caesarean section were excluded from analysis (*N* = 18). In 19 cases, data were missing on covariates such as level of education (*N* = 8), parity (*N* = 6), vaginal tear (*N* = 3), and depressive symptoms at the time of completing the questionnaire measuring childbirth experience (*N* = 4). These women were also excluded from analyses, resulting in a final sample of 486 women in the current study. Table 1 shows the characteristics of the participating women.

**Table 1:** Characteristics of the participating women (*N* = 486)

	*N*	%	Mean (SD)	Range
*Maternal characteristics*				
Age			30.2 (3.5)	20–43
High level of education	335	68.9		
High SES	275	56.6		
Paid job	450	93.0		
Living with a partner	482	99.2		
Depression earlier in life	70	14.5		
*Obstetric features*				
Multiparity	241	49.6		
Gestational age at delivery (weeks)			39.9 (1.2)	35–41
Premature birth (≤ 37 weeks)	10	2.1		
Mode of delivery				
Spontaneous delivery	273	56.2		
Non-spontaneous delivery				
Induced labor	144	29.6		
Vacuum-/forceps-assisted delivery	36	7.4		
Secondary Caesarean section	33	6.8		
Episiotomy	164	33.7		
Vaginal tear	51	10.5		

*SD*, standard deviation; *High level of education*, bachelor’s or master’s degree; *High SES*, socioeconomic status 4–6; *Vaginal* *tear*, second degree or third degree (total rupture)

### Measures

#### Mindfulness during pregnancy

Trait mindfulness was measured at 22 weeks of pregnancy using the Dutch version of the 12-item Three Facet Mindfulness Questionnaire-Short Form (TFMQ-SF) (Truijens et al. 2016). The TFMQ-SF was developed based on the short form of the Five Facet Mindfulness Questionnaire (FFMQ) (Baer et al. 2006; Bohlmeijer et al. 2011). It consists of three subscales, each containing four items and measuring a different facet of mindfulness: (1) *acting with awareness*, which is the opposite of acting on automatic pilot, (2) *non-judging* of one’s thoughts and feelings, but rather being accepting towards them, and (3) *non-reacting* to one’s disturbing thoughts and feelings. The total score per subscale ranges from 4 to 20, with higher scores indicating greater levels of mindfulness. The three subscales of the TFMQ-SF have shown to be reliable and valid in Dutch pregnant women, with Cronbach’s alphas of 0.87 (*acting with awareness*), 0.84 (*non-judging*) and 0.81 (*non-reacting*) (Truijens et al. 2016). The Cronbach’s alphas in the current study were 0.86, 0.80, and 0.80, respectively.

#### Perception of childbirth

Perception of childbirth was assessed between 7 and 21 days postpartum, using the six-item Perception of Delivery (PD) subscale of the Childbirth Perception Scale (CPS) (Truijens et al. 2014b). Examples of items are “My labor was a lot worse than I expected” and “I panicked during my labor”. Total scores range from 0 to 24 with higher scores reflecting a more negative perception of childbirth. The CPS has been validated in Dutch postpartum women, showing adequate psychometric properties with a Cronbach’s alpha of 0.81 for the PD subscale (Truijens et al. 2014b). In the current study, Cronbach’s alpha was 0.77.

#### Depressive symptoms at the time of completing the CPS

Depressive symptoms over the past 7 days were assessed using the Dutch version of the 10-item Edinburgh Postnatal Depression Scale (EPDS) (Cox et al. 1987; Pop et al. 1992). Women completed the EPDS at the same time as the CPS (between 7 and 21 days postpartum) since depressive symptoms can influence the recollection of experiences (Burt et al. 1995; Matt et al. 1992). Total EPDS scores range from 0 to 30 with higher scores indicating higher levels of depressive symptoms. The EPDS has been validated in Dutch postpartum women and has appropriate psychometric properties with a Cronbach’s alpha of 0.82 (Pop et al. 1992). In the current study, Cronbach’s alpha was 0.86.

#### Covariates

Demographic characteristics were obtained by questionnaires at baseline: 12 weeks of pregnancy, including *age*, *level of education* (low or medium/high (high = bachelor’s or master’s degree)), *socioeconomic status (SES)* (low or medium = 1–3/high = 4–6), having a *paid job* (yes/no), *living with a partner* (yes/no), *depressive episode earlier in life* (yes/no), and *parity* (primiparous/multiparous). Obstetric data were extracted from the obstetric records, such as *mode of delivery* (spontaneous delivery/non-spontaneous delivery (non-spontaneous delivery = induced labor, vacuum- or forceps-assisted delivery, secondary Caesarean section)), *episiotomy* (yes/no), *vaginal tear* (none or first degree/second degree or third degree (total rupture)), and *gestational age at birth* (weeks).

### Statistical analysis

Potential differences between the women included in the current study and the remainder of the HAPPY sample were assessed using independent *t* tests and chi-square tests. Pearson *r* correlations were computed between the three facets of mindfulness during pregnancy, perception of childbirth, and depressive symptoms at the time of completing the CPS. Effect sizes were calculated with regard to Cohen’s *d* (0.20 = small, 0.50 = medium, and 0.80 = large) and *r* (0.10 = small, 0.30 = medium, and 0.50 = large) (Cohen 1988).

Next, several multiple linear regression analyses were performed, with perception of childbirth as the dependent variable to assess a possible direct association between maternal dispositional mindfulness and subjective childbirth experience. In the first step, the three facets of mindfulness during pregnancy were entered together into the regression. Thereafter, the following seven covariates were entered: level of education, SES, parity, depressive symptoms at the time of completing the CPS, episiotomy, vaginal tear, and non-spontaneous delivery, resulting in a model that included ten predictors.

Subsequently, Hayes’ SPSS PROCESS macro 3.4 was used to examine the potential moderating effect of trait mindfulness during pregnancy on the possible association between non-spontaneous delivery and perception of childbirth, to examine whether trait mindfulness protects against the adverse effects of a non-spontaneous delivery. Simple moderation with mean centering was used to analyze each facet of mindfulness separately as moderator, while the other two facets were included in the model as covariates. The other six covariates included in the model were level of education, SES, parity, depressive symptoms at the time of completing the CPS, episiotomy, and vaginal tear. This resulted in three possible models, all including ten predictors and one interaction. When a significant interaction (*p* < .05) occurred, the PROCESS macro examined conditional effects of the independent variable (non-spontaneous delivery) on the dependent variable (perception of childbirth) at three levels (16th, 50th, and 84th percentiles) of the moderator (a facet of mindfulness during pregnancy), to interpret the findings. The 16th, 50th, and 84th percentiles were used to guarantee that the probed points were within the observed range of the data (Hayes 2018).

## Results

First, characteristics of the women in the current study (*N* = 486) were compared with that of the sample who did not complete the mindfulness assessment during pregnancy or the perception of delivery assessment between 7 and 21 days postpartum (*N* = 1783). The current sample was more often highly educated (*p* = .002, phi coefficient = 0.07, small effect size), differed in gestational age at delivery (*M* = 39.9 and *M* = 39.4 respectively, *p* < .001, Cohen’s *d* = 0.25, small effect size) and included fewer preterm births (*p* < .001, phi coefficient = 0.12, small effect size). Furthermore, the women in the current study scored significantly higher on the non-reacting facet of mindfulness (*M* = 11.9 and *M* = 11.2 respectively, *p* = .007, Cohen’s *d* = 0.18, small effect size). The two samples did not differ regarding acting with awareness and non-judging, depressive symptoms at the time of completing the CPS, age, SES, paid job, living with a partner, history of depressive episode, parity, mode of delivery, vaginal tear and episiotomy.

Table 2 shows the Pearson *r* correlations between the three facets of mindfulness during pregnancy, perception of childbirth, and depressive symptoms at the time of completing the CPS. The mindfulness facets acting with awareness and non-reacting were significantly inversely associated with the perception of childbirth (acting with awareness, *r* = − 0.092, *p* = .042; non-reacting, *r* = − 0.133, *p* = .003; both small effect sizes).

**Table 2:** Correlations between facets of mindfulness during pregnancy, perception of childbirth, and depressive symptoms at the time of completing the CPS, mean scores, and ranges (*N* = 486)

	1	2	3	4	5
1. TFMQ-SF: acting with awareness	1	0.401***	− 0.273***	− 0.092*	− 0.164***
2. TFMQ-SF: non-judging	-	1	− 0.208***	− 0.070	− 0.342***
3. TFMQ-SF: non-reacting	-	-	1	− 0.133**	− 0.030
4. CPS (PD): perception of childbirth	-	-	-	1	0.331***
5. EPDS: depressive symptoms at the time of completing the CPS	-	-	-	-	1
Mean (SD)	14.5 (3.0)	16.2 (3.1)	11.9 (4.1)	5.1 (3.2)	4.8 (4.8)
Range	7–20	7–20	4–20	0–15	0–28

*TFMQ-SF*, Three Facet Mindfulness Questionnaire-Short Form (22 weeks of pregnancy); *CPS*, Childbirth Perception Scale; *PD*, Perception of Delivery subscale (7–21 days after delivery), higher scores reflect a more negative perception of childbirth; *EPDS*, Edinburgh Postnatal Depression Scale (7–21 days after delivery); *SD*, standard deviation

*p < .05 (two-tailed)

**p < .01 (two-tailed)

***p < .001 (two-tailed)

The regression models had variance inflation factor values < 2, indicating no signs of multicollinearity. In the first step of the linear regression analysis (see Table 3, model 1), 3.9% of the variance was explained by the three facets of mindfulness and the model was significant (*F*(3, 482) = 8.43, *p* < .001). Acting with awareness (*B* = − 0.13, *p* = .020) and non-reacting (*B* = − 0.14, *p* < .001) were significantly negatively associated with the perception of childbirth. After adjusting for the level of education, SES, parity, depressive symptoms at the time of completing the CPS, episiotomy, vaginal tear and non-spontaneous delivery, acting with awareness, and non-reacting still remained significant predictors of perception of childbirth (acting with awareness, *B* = − 0.11, *p* = .033; non-reacting, *B* = − 0.08, *p* = .034).

**Table 3:** Multiple regression predicting the perception of childbirth (*N* = 486)

	*B* (SE)	*T*	95% CI
Model 1^a^			
TFMQ-SF: acting with awareness	− 0.13 (0.05)	− 2.34*	− 0.23, − 0.02
TFMQ-SF: non-judging	− 0.06 (0.05)	− 1.22	− 0.16, 0.04
TFMQ-SF: non-reacting	− 0.14 (0.04)	− 3.79***	− 0.21, − 0.07
Model 2^b^			
TFMQ-SF: acting with awareness	− 0.10 (0.05)	− 2.12*	− 0.20, − 0.01
TFMQ-SF: non-judging	0.07 (0.05)	1.46	− 0.03, 0.17
TFMQ-SF: non-reacting	− 0.07 (0.04)	− 2.04*	− 0.14, − 0.003
High level of education	− 0.54 (0.31)	− 1.74^†^	− 1.16, 0.07
High SES	0.07 (0.24)	0.29	− 0.40, 0.55
Multiparity	0.12 (0.29)	0.41	− 0.45, 0.69
Depressive symptoms at the time of completing the CPS	0.20 (0.03)	6.40***	0.14, 0.26
Episiotomy	0.88 (0.30)	2.90**	0.28, 1.48
Vaginal tear	1.04 (0.44)	2.38*	0.18, 1.89
Non-spontaneous delivery	1.45 (0.28)	5.12***	0.90, 2.01
Non-spontaneous delivery * TFMQ-SF: acting with awareness	− 0.19 (0.09)	− 2.18*	− 0.37, − 0.02
Model 3^c^			
TFMQ-SF: acting with awareness	− 0.10 (0.05)	− 1.94^†^	− 0.19, 0.001
TFMQ-SF: non-judging	0.05 (0.05)	1.10	− 0.04, 0.15
TFMQ-SF: non-reacting	− 0.08 (0.04)	− 2.21*	− 0.15, − 0.01
High level of education	− 0.51 (0.31)	− 1.65	− 1.13, 0.10
High SES	0.09 (0.24)	0.38	− 0.38, 0.57
Multiparity	0.11 (0.29)	0.37	− 0.46, 0.68
Depressive symptoms at the time of completing the CPS	0.19 (0.03)	6.31***	0.13, 0.25
Episiotomy	0.87 (0.30)	2.86**	0.27, 1.47
Vaginal tear	1.09 (0.43)	2.51*	0.24, 1.94
Non-spontaneous delivery	1.45 (0.28)	5.10***	0.89, 2.01
Non-spontaneous delivery * TFMQ-SF: non-judging	− 0.18 (0.09)	− 2.10*	− 0.35, − 0.01

*Perception of childbirth*, higher scores reflect a more negative perception of childbirth; *B*, unstandardized regression coefficient; *SE*, standard error; *CI*, confidence interval; *TFMQ-SF*, Three Facet Mindfulness Questionnaire-Short Form; *SES*, socioeconomic status; *CPS*, Childbirth Perception Scale

^a^Model 1, three facets of Three Facet Mindfulness Questionnaire-Short Form

^b^Model 2, adjusted for possible covariates and moderator variable acting with awareness

^c^Model 3, adjusted for possible covariates and moderator variable non-judging

^†^*p* < .10

*p < .05

**p < .01

***p < .001

Moderation analyses of each facet of mindfulness showed a significant interaction between acting with awareness and non-spontaneous delivery (*B* = − 0.19, *p* = .030). Table 3 model 2 shows the linear regression model adjusted for covariates (non-judging, non-reacting, level of education, SES, parity, depressive symptoms at the time of completing the CPS, episiotomy, and vaginal tear), including the interaction between acting with awareness and non-spontaneous delivery. The total model was significant (*F*(11, 474) = 12.59, *p* < .001) and explained 22.6% of the variance. The addition of the interaction was a significant change to the model (*F*(1, 474) = 4.74, *p* = .030, *R*^2^ change = 0.008). In addition to the moderating effect of acting with awareness, both acting with awareness and non-reacting were significant predictors of perception of childbirth (acting with awareness, *B* = − 0.10, *p* = .034; non-reacting, *B* = − 0.07, *p* = .042).

Moreover, a significant interaction between non-judging and non-spontaneous delivery was found (*B* = − 0.18, *p* = .036). The linear regression model, including the interaction between non-judging and non-spontaneous delivery, adjusted for covariates (acting with awareness, non-reacting, level of education, SES, parity, depressive symptoms at the time of completing the CPS, episiotomy and vaginal tear), is shown in Table 3, model 3. The model explained 22.6% of the variance and was significant (*F*(11, 474) = 12.55, *p* < .001). The addition of the interaction was a significant change to the model (*F*(1, 474) = 4.30, *p* = .036, *R*^2^ change = 0.007). In addition to the moderating effect of non-judging, non-reacting remained significantly associated with perception of childbirth (*B* = − 0.08, *p* = .028), and acting with awareness was significantly associated with perception of childbirth at 90% level (*B* = − 0.10, *p* = .053).

**Fig. 1:**
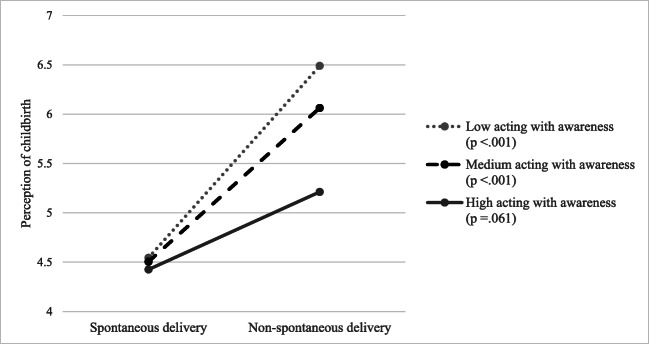
Association between (non-)spontaneous delivery and perception of childbirth (higher scores reflect a more negative perception of childbirth) at three levels of the mindfulness facet acting with awareness (low, 16th percentile; medium, 50th percentile; high, 84th percentile) (*N* = 486)

Figure 1 shows the association of non-spontaneous delivery with perception of childbirth at three levels of acting with awareness. Simple slopes were tested across the three levels of acting with awareness. Only for low and medium scores of acting with awareness did non-spontaneous delivery predict a more negative perception of childbirth (low acting with awareness, *B* = 1.95, *p* < .001; medium acting with awareness, *B* = 1.56, *p* < .001; high acting with awareness, *B* = 0.79, *p* = .062).

**Fig. 2:**
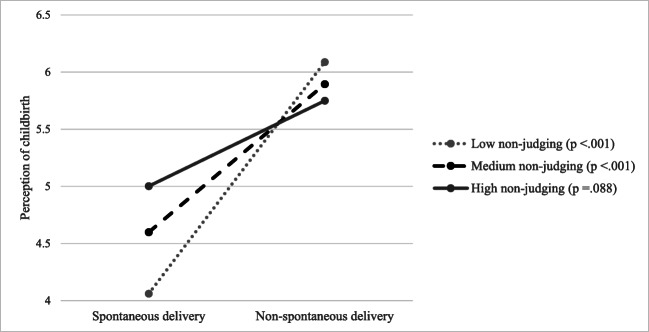
Association between (non-)spontaneous delivery and perception of childbirth (higher scores reflect a more negative perception of childbirth) at three levels of the mindfulness facet non-judging (low, 16th percentile; medium, 50th percentile; high, 84th percentile) (*N* = 486)

The association of non-spontaneous delivery with perception of childbirth at three levels of non-judging is shown in Fig. 2. Non-spontaneous delivery predicted a more negative perception of childbirth for low and medium scores of non-judging (low non-judging, *B* = 2.03, *p* < .001; medium non-judging, *B* = 1.30, *p* < .001; high non-judging, *B* = 0.75, *p* = .093).

Repeated analyses excluding 59 women with antenatal depression (EDS ≥ 10) at 22 weeks of pregnancy showed similar results. In this sample (*N* = 427), acting with awareness and non-reacting remained significant predictors of perception of childbirth (acting with awareness, *B* = − 0.12, *p* = .029; non-reacting, *B* = − 0.08, *p* = .035). Moderation analyses showed a significant interaction between acting with awareness and non-spontaneous delivery at 90% level (*B* = − 0.19, *p* = .059) and a significant interaction between non-judging and non-spontaneous delivery (*B* = − 0.27, *p* = .010).

## Discussion

The current study showed that several aspects of maternal dispositional mindfulness were associated with a more positive postnatal perception of the mother’s most recent childbirth (even after the exclusion of women with antenatal depression). Regression analyses showed that the mindfulness facets acting with awareness and non-reacting were significantly associated with a more positive perception of childbirth, after adjustment for potential confounders (level of education, SES, parity, depressive symptoms at the time of completing the CPS, episiotomy, vaginal tear and non-spontaneous delivery). Moderation analyses showed a significant interaction between the mindfulness facet acting with awareness and non-spontaneous delivery and the mindfulness facet non-judging and non-spontaneous delivery in predicting perception of childbirth.

The regression models accounted for a significant proportion of the variance in perception of childbirth. It must be noted that trait mindfulness only explained a small proportion of the variance, in both the direct association and moderating association. This small magnitude should be taken into account when interpreting the results. Most of the variance in the models was explained by depressive symptoms at the time of completing the CPS, with similar results after exclusion of women with antenatal depression. Indeed, depression has been associated with memory impairment (Burt et al. 1995). Individuals with depressive symptoms tend to recollect negative experiences better than positive experiences (Matt et al. 1992). Therefore, our results could imply that the recollection of the childbirth experience at the time of completing the CPS was considerably influenced by the level of depressive symptoms.

Nonetheless, the findings of the current study suggest that trait mindfulness during pregnancy may enhance a positive childbirth experience. Without an intervention, trait mindfulness appears to be stable over time (Brown and Ryan 2003). Therefore, it can be presumed that the trait mindfulness scores were relatively stable in the current study, between measurement of mindfulness at 22 weeks of pregnancy and perception of childbirth between 7 and 21 days postpartum, since the women did not partake in a mindfulness intervention. The results of the current study are in line with our expectations. Even though there are no quantitative studies assessing this direct association between trait mindfulness and childbirth perception, the results of a previously conducted qualitative intervention study showed that women who participated in antenatal mindfulness-based childbirth education managed to maintain feelings of control, even when the childbirth did not proceed as planned (Fisher et al. 2012). In turn, feelings of control during childbirth have been related to a more positive childbirth experience (Waldenstrom 1999; Waldenstrom et al. 2004).

In the current study, higher levels of acting with awareness were shown to be associated with a more positive perception of childbirth. Acting with awareness can be described as being attentive to experiences in the present moment (Brown and Ryan 2003). While the overwhelming nature of childbirth imposes a risk for avoidance and dissociation during childbirth (Byrne et al. 2017), being able to stay present with the birthing process may help women cope with the intensity and pain of the labor. In a qualitative study (Whitburn et al. 2014), women were interviewed on how they experienced labor pain, and two distinctive states of mind were found. The first one, which was associated with a more positive birth experience, was characterized not only by a focused attention but also by openness and acceptance of all experiences. On the other hand, the second state of mind, which was associated with a less positive birth experience, was characterized by distraction. Keeping the attention focused in the present moment may help prevent women from catastrophizing, which is considered as an unhelpful coping strategy (Escott et al. 2009; Escott et al. 2004). Indeed, the second state of mind described in the study by Whitburn et al. (2014) was characterized by pain catastrophizing and a negative evaluation of pain. Being attentive to experiences in the present moment may also decrease the risk for avoidance and dissociation, which have shown to be associated with the development of a posttraumatic stress disorder in general (Ozer et al. 2003). In this interpretation, it is important to take into account that we presume that trait mindfulness, measured at 22 weeks of pregnancy, is a relatively stable factor and that women who had a high score on acting with awareness during pregnancy, could also rely on this during childbirth. However, the extent to which mindfulness during pregnancy is predictive of the extent to which women are also mindful during childbirth was not investigated.

Acting with awareness not only was shown to be associated with a more positive childbirth experience in general but also moderated the association between non-spontaneous delivery and a more negative perception of childbirth. In women who scored high on acting with awareness, non-spontaneous delivery was not associated with a more negative childbirth experience, which suggests that acting with awareness may serve as a protective factor against the adverse effects of a non-spontaneous delivery. Non-spontaneous delivery is associated not only with physical but also psychological consequences (Rowlands and Redshaw 2012), such as feelings of dissatisfaction about the delivery (Shetty et al. 2005). Possibly, for women with a high level of acting with awareness, it may be easier to bring back their attention to their baby and caring for their baby when thoughts arise regarding the delivery.

The mindfulness facet non-reacting refers to allowing thoughts and feelings to come and go, without getting carried away by them. This is the opposite of worrying and rumination (Baer et al. 2006), and in the general population, non-reacting is negatively associated with symptoms of depression and anxiety (Bohlmeijer et al. 2011; Branstrom et al. 2011). However, for pregnant women, non-reacting may be less appropriate and helpful. Usually, pregnant women undergo an intrapsychic change that is called the motherhood constellation (Stern 1995), which means that the mother becomes pre-occupied with protecting her child. This preoccupation is a healthy adjustment to the changes that are and will occur regarding the emotional investment in the baby, needed to fulfill the baby’s needs. Thus, for pregnant women, the ability to let go of thoughts about the pregnancy and the (unborn) baby may not be emotionally healthy, which may explain the negative correlation with the other facets of mindfulness and the absent correlation with symptoms of depression. Nevertheless, higher levels of non-reacting were found to be associated with a more positive perception of childbirth. This could imply that when women score higher on non-reacting during pregnancy, they worry less about birth-related matters and have a more positive childbirth experience. Indeed, worrying about the birth in early pregnancy has been associated with a more negative childbirth experience (Waldenstrom 2004). Also, non-reacting may support women in labor in being able to let go of unpleasant thoughts, reducing the aversive aspects of the experience. This may contribute to accepting pain as an inevitable part of childbirth, which is considered as a helpful coping strategy (Van der Gucht and Lewis 2015). Non-reacting did not moderate the association between non-spontaneous birth and childbirth experience. Thus, non-reacting was important in the birth process for all women and did not play an extra protective role for women with a non-spontaneous delivery.

The mindfulness facet non-judging was not found to be directly associated with perception of childbirth. Non-judging involves accepting thoughts and feelings without judgment or self-criticism (Baer et al. 2006). Non-judging did however moderate the association between non-spontaneous delivery and a more negative perception of childbirth. In women who scored high on non-judging, non-spontaneous delivery was not associated with a more negative childbirth experience, which means that non-judging may serve as a protective factor against the adverse effects of a non-spontaneous delivery. Non-judging may be especially important for women who experienced a non-spontaneous delivery, because these women are at risk for negative perceptions of themselves (Lobel and DeLuca 2007), and “feeling like a failure” (Carquillat et al. 2016; Kjerulff and Brubaker 2018). According to a qualitative study by Guittier et al. (2014), mode of delivery influences women’s self-worth and self-confidence regarding motherhood. Possibly, women with a high level of non-judging are less self-critical in the period after the non-spontaneous birth and therefore look back at the delivery less negatively compared with women with lower levels of non-judging.

Previous research also showed that mindfulness moderated the association between unavoidable distressing events and adverse effects on mental health (Bergomi et al. 2013a). Moreover, acting with awareness, non-judging, and non-reacting were found to moderate the association between stressful life events and depression and anxiety in adults with diabetes (van Son et al. 2015). In adults with rheumatoid arthritis, mindfulness seemed to prospectively buffer against negative associations between disability and psychological distress (Nyklíček et al. 2015). Furthermore, mindfulness has been found to moderate the effects of laboratory stress tests on cortisol and affective responses (Brown et al. 2012). These results correspond with the potential buffering role of the two facets of mindfulness in the current study, since childbirth could be a stressful life event, especially when the delivery is more complicated.

The strengths of the current study include the longitudinal design and large sample size. Limitations include the use of self-report measures only for the assessment of mindfulness (Truijens et al. 2016) and depressive symptoms at the time of completing the CPS (Cox et al. 1987; Pop et al. 1992). A diagnostic psychiatric interview with which it is possible to assess syndromal depression was not used. Also, self-report assessment in general could introduce bias due to social desirability and personal values, which is specifically relevant in mindfulness assessment (Bergomi et al. 2013b). Moreover, instead of asking women whether they were ever diagnosed with depression (yes/no), a lifetime prevalence interview (CIDI, SCID) would have been more accurate. However, due to the large sample size of the study, structured interviews were not affordable to be performed.

Furthermore, the measurement of trait mindfulness at 22 weeks of pregnancy was a limitation. We presumed that trait mindfulness scores were relatively stable over time in the current study and that women with high mindfulness scores during pregnancy could also rely on this during childbirth. Besides conceptualizing mindfulness as a trait, mindfulness could also be seen as a state, which is more situation-specific and variable over time (Bishop et al. 2004; Tanay and Bernstein 2013), meaning that mindfulness measured at 22 weeks of pregnancy was not necessarily associated with mindfulness during and after childbirth. However, we conceptualized mindfulness as a trait in the current study, and since a test-retest reliability in a Dutch, mostly female, sample showed good stability of mindfulness across time (Veehof et al. 2011), it is likely that there was no substantial change in trait mindfulness scores, between the measurement of maternal dispositional mindfulness at 22 weeks of pregnancy and perception of childbirth between 7 and 21 days postpartum.

Furthermore, social support during childbirth was not measured, as well as subjective experiences of previous childbirths, and we were therefore unable to include these variables as covariates in our regression models. A Cochrane review concluded that women, who had continuous support from a doula, partner, family member, or friend during the delivery, were more likely to have a spontaneous vaginal birth and a shorter labor, were less likely to have a Caesarean birth or instrumental vaginal birth, and were less likely to report negative ratings or feelings about their birth experience (Bohren et al. 2017). These results suggest that continuous social support could be a possible protective factor for the negative perception of childbirth. Future research should address social support during childbirth and its associations with the perception of childbirth.

Moreover, generalizability of the findings is limited because Dutch mostly white and highly educated women were involved, different from the national figures (Statistics 2019). In the current sample, the variability of the included demographic factors, such as high level of education and high SES, could have been too small to show a significant association with perception of childbirth. Women of ethnic minorities more often have poorer birth outcomes (MacDorman 2011; Thompson and Suter 2020), also limiting the generalizability of the results of the current study, since the sample only included women of one race.

In conclusion, the results of the current study suggest that trait mindfulness during pregnancy may enhance a positive perception of childbirth and could serve as a protective factor when the delivery is more complicated. Because of the small effect sizes, the results should be interpreted with caution. These findings are a first indication that maternal dispositional mindfulness is associated with a more positive childbirth perception. Throughout time, maternal dispositional mindfulness can be improved by practicing state mindfulness during mindfulness meditation (Kiken et al. 2015). Therefore, a possibly important clinical implication of our findings is that mindfulness-based programs during pregnancy could be helpful in enhancing the subjective childbirth experience. Compared with our findings, increasing trait mindfulness across time could perhaps provide a larger positive effect on childbirth perception. Mindfulness-based interventions are usually well received, and the adherence has been described as acceptable (Lever Taylor et al. 2016). Previous studies reported positive results of antenatal mindfulness-based interventions regarding the use of mindfulness skills during delivery (Duncan and Bardacke 2010; Lonnberg et al. 2018). Literature assessing the effectiveness with regard to childbirth perception is however scarce and future research is needed. The effectiveness of different types of mindfulness-based programs during pregnancy should be examined. For example, pregnant women could partake in an 8-week Mindfulness-Based Stress Reduction (MBSR) program (Segal et al. 2013) to practice mindfulness meditation in general. Moreover, a 9-week Mindfulness-Based Childbirth and Parenting (MBCP) program could be useful. MBCP is a childbirth education program that integrates mindfulness meditation with the knowledge of psychobiological processes in pregnancy, childbirth, and early parenting (Duncan and Bardacke 2010). Providing a mindfulness-based intervention by means of the internet could reduce costs and increase accessibility. A recent study found promising effects of an online mindfulness-based intervention for pregnant women but showed low adherence to the intervention (Krusche et al. 2018). Therefore, it is important to examine ways to increase the acceptability of online mindfulness-based interventions. Furthermore, mindfulness meditation could be incorporated in childbirth education, such as in the 8-week antenatal mindfulness-based childbirth education (MBCE). This intervention combines MBSR with skills-based childbirth education and helps women using mindfulness in discomforting situations during pregnancy, labor, and parenting (Hauck et al. 2016). It is important to examine whether mindfulness-based programs that focus specifically on pregnant women’s needs and preparation for childbirth would be more effective in enhancing childbirth perception compared with general programs such as MBSR, to give pregnant women the opportunity to choose the program that is most evidence-based. When proven effective, future research should roll out strategies for implementing these programs in perinatal healthcare.

Furthermore, future research is needed to examine whether the results of the current study can be confirmed in other, more diverse, samples. This could provide insight into possible cultural variation in maternal dispositional mindfulness. It could also address possible cultural differences in the effects of trait mindfulness on perception of childbirth and the extent to which trait mindfulness may protect against the adverse effects of a more complicated delivery. Moreover, it is important to examine cultural differences in acceptability and effectiveness of antenatal mindfulness-based childbirth education and (online) mindfulness-based interventions. Previous research found promising results of mindfulness-based interventions in ethnic minorities but recommends adaptations of these programs to align with the cultural values of particular communities (Nagayama Hall et al. 2011; Woods-Giscombe and Gaylord 2014). Therefore, future research should especially focus on the acceptability and effectiveness of different cultural-specific programs.

Finally, enhancing a positive childbirth experience is important in reducing levels of postpartum depression and childbirth-related posttraumatic stress symptoms and their negative consequences for mother and child. Future research should address possible associations between maternal mindfulness and these maternal mental health challenges.
